# Supplementary material for: Serine peptidases and increased amounts of soluble proteins contribute to heat priming of the plant pathogenic fungus Botrytis cinerea
Source: mBio. 2023 Jul 6;14(4):e01077-23. doi: 10.1128/mbio.01077-23 (PMC10470532; doi:10.1128/mbio.01077-23)
Supplement: Fig. S1 — Growth responses of B. cinerea to different temperatures. [file mbio.01077-23-s0001.pdf]

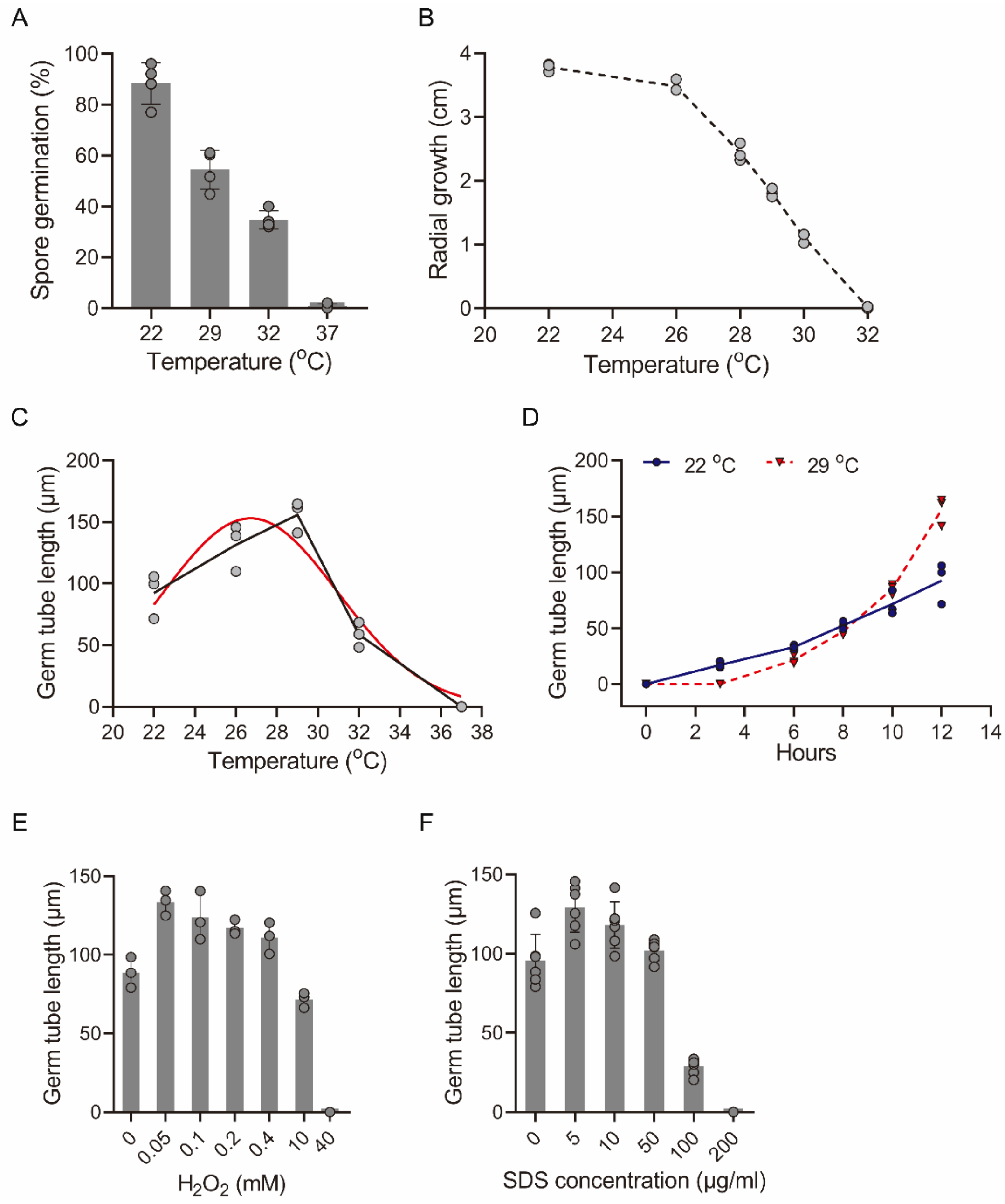

Fig S1. Growth responses of *B. cinerea* to different temperatures**.** (A) Spore germination. Spores were incubated at the indicated temperature, and germination rates were determined after 3 h of incubation at 22°C, 4 h at 29°C, 8 h at 32°C or 12 h at 37°C. (B) Colony growth. Colonies were initiated from mycelial plugs, and radial growth was measured after 3 d of incubation at the indicated temperatures. (C) Germ tube growth. Spores were incubated at the indicated temperatures, and GT length was measured after 12 hours of incubation. Red line shows a Gaussian regression curve (R^2 = 0.9186). (D) Germ tube length over time. Spores were incubated at 22°C or 29°C, and GT length was recorded at different time intervals. (E, F) Effects of oxidative (H_2O_2) and cell wall (SDS) stress on GT growth. Spores were incubated in medium with the indicated concentrations of H_2O_2 or SDS, and GT length was recorded after 12 h. Graphs represent

four (A), three (B–E) and six (F) biological replications with overlaid individual data points. For A, E and F, values are presented as the mean of replicates ± s.d.
