# Supplementary material for: Serine peptidases and increased amounts of soluble proteins contribute to heat priming of the plant pathogenic fungus Botrytis cinerea
Source: mBio. 2023 Jul 6;14(4):e01077-23. doi: 10.1128/mbio.01077-23 (PMC10470532; doi:10.1128/mbio.01077-23)
Supplement: Fig. S2 — Effects of priming on fungal survival, recovery, and pathogenicity. [file mbio.01077-23-s0002.pdf]

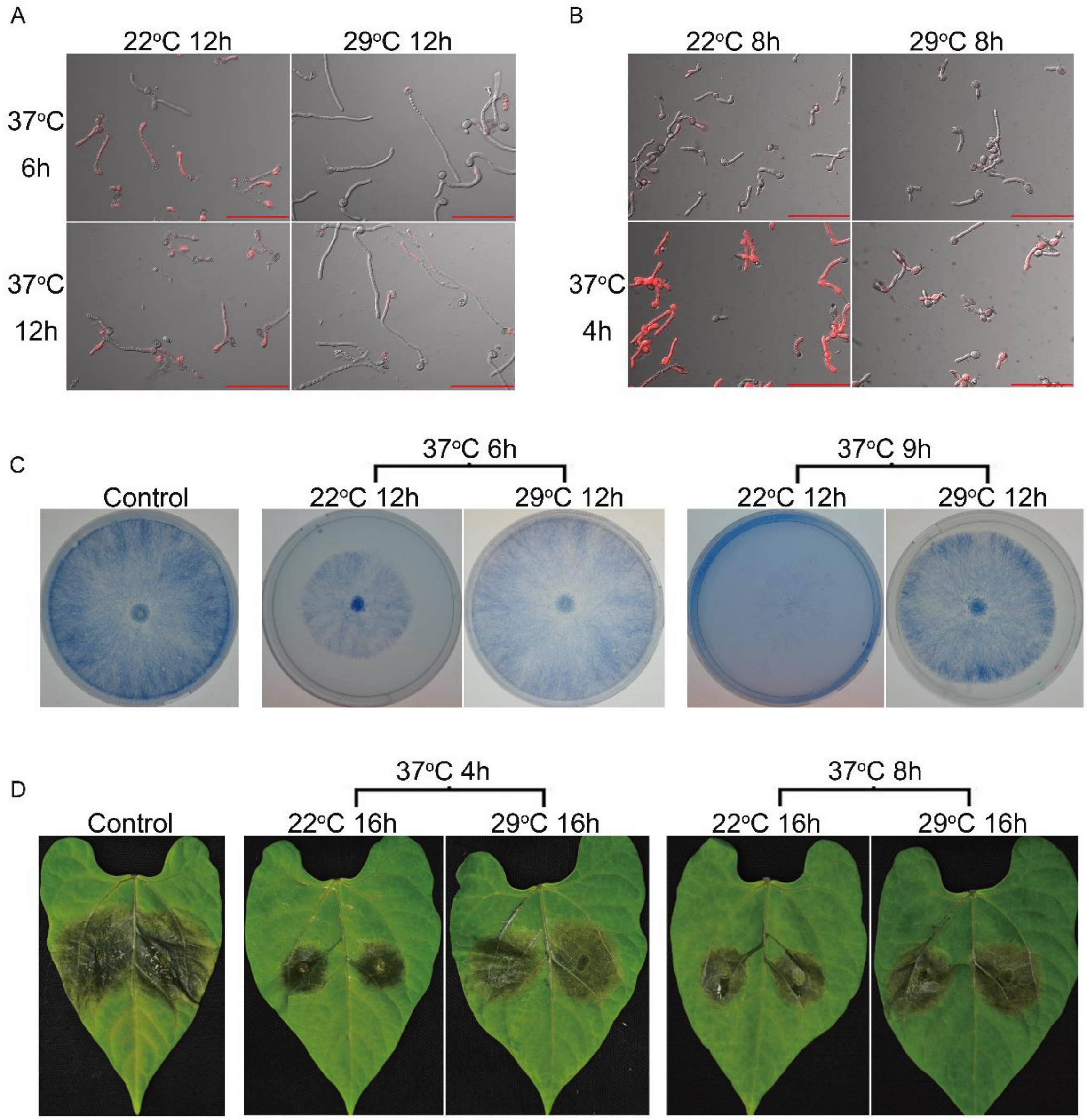

Fig S2. Effects of priming on fungal survival, recovery, and pathogenicity. (A, B) Combined DIC and fluorescence (rhodamine filter) microscopic images of GTs after staining with PI (A) or DiBAC4(5) (B). Scale bars, 100 µm. (C) Recovery growth of fungal cultures after exposure to 37°C with and without priming. Cultures were stained with cotton blue for better visualization of mycelia. (D) Infection symptoms of inoculated French bean (*Phaseolus vulgaris*) leaves following exposure to 37°C with and without priming.
