# Supplementary material for: Serine peptidases and increased amounts of soluble proteins contribute to heat priming of the plant pathogenic fungus Botrytis cinerea
Source: mBio. 2023 Jul 6;14(4):e01077-23. doi: 10.1128/mbio.01077-23 (PMC10470532; doi:10.1128/mbio.01077-23)
Supplement: Fig. S3 — RNA-seq data. [file mbio.01077-23-s0003.pdf]

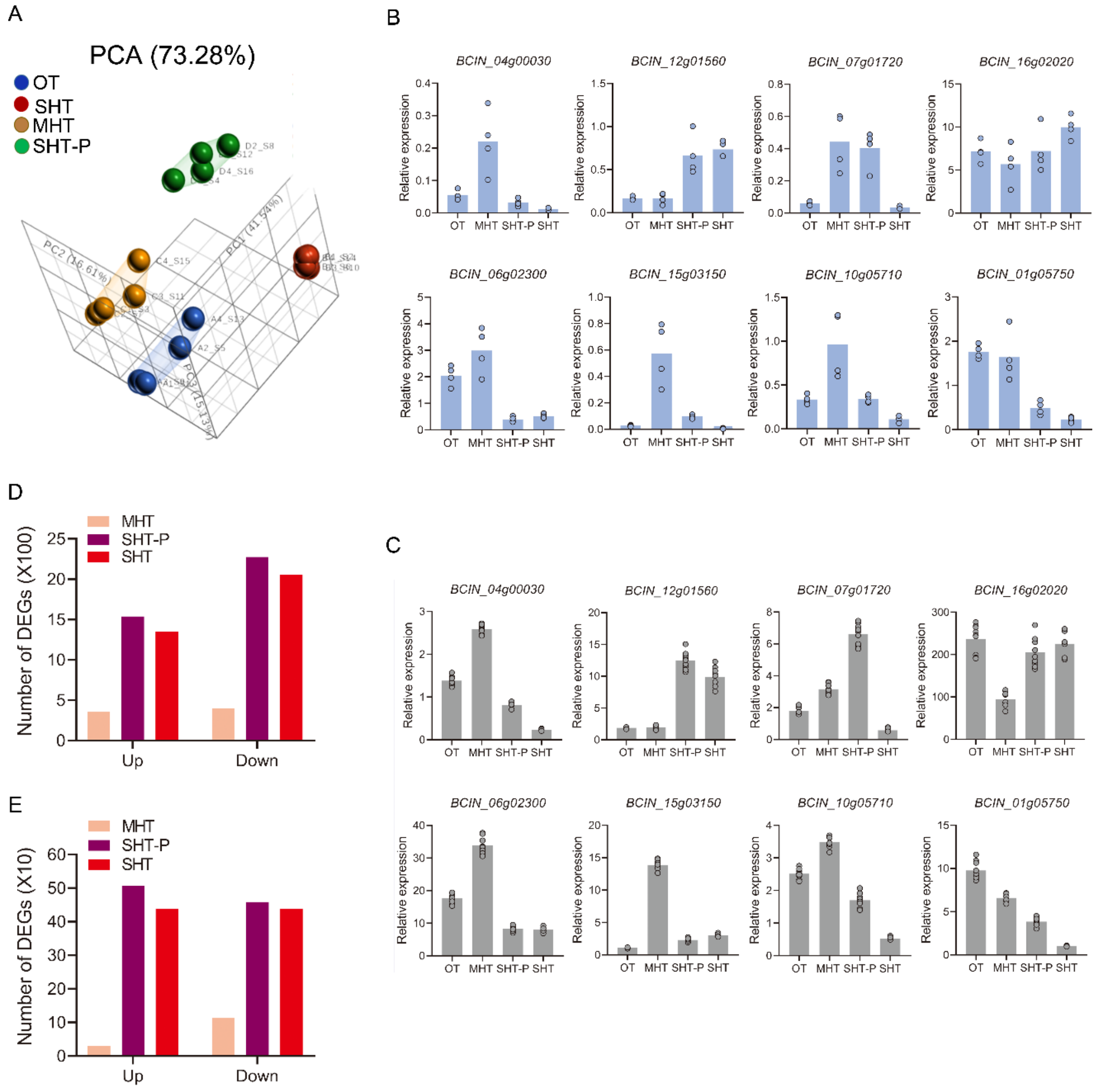

Fig S3. RNA-seq data. (A) Principal component analysis (PCA) of all genes that were identified by RNA-seq. Fungi were grown under optimal temperature (OT, 22°C), moderately high temperature (MHT, 29°C), severely high temperature (SHT, 22°C + 37°C) or severely high temperature conditions with priming (SHT-P, 29°C + 37°C). Mycelia were harvested, and RNA was extracted and sequenced in one lane of the Illumina NextSeq 500 flow cell using the 75-bp single-end RNA-seq protocol. (B) Relative expression levels of eight genes in the RNA-seq data. Relative expression was calculated using the RPKM value under each treatment (four biological replications) and normalized to the expression of the ribosomal protein gene *bcrsm18*. Graphs represent four biological replications with overlaid individual data points. (C) Validation of the RNA-seq results by RT-PCR analysis. Relative expression was normalized to the ribosomal protein gene *bcrsm18*. Graphs represent nine overlaid individual data points from three independent biological replicates and three technical replicates. (D) Number of DEGs (pFDR < 0.05, lFCl ≥ 2) at MHT, SHT-

P and SHT compared to OT. (E) Number of DEGs (pFDR < 0.05, lFCl ≥ 5) at MHT, SHT-P and SHT compared to OT.
