# Supplementary material for: Serine peptidases and increased amounts of soluble proteins contribute to heat priming of the plant pathogenic fungus Botrytis cinerea
Source: mBio. 2023 Jul 6;14(4):e01077-23. doi: 10.1128/mbio.01077-23 (PMC10470532; doi:10.1128/mbio.01077-23)
Supplement: Fig. S4 — Proteomic data. [file mbio.01077-23-s0004.pdf]

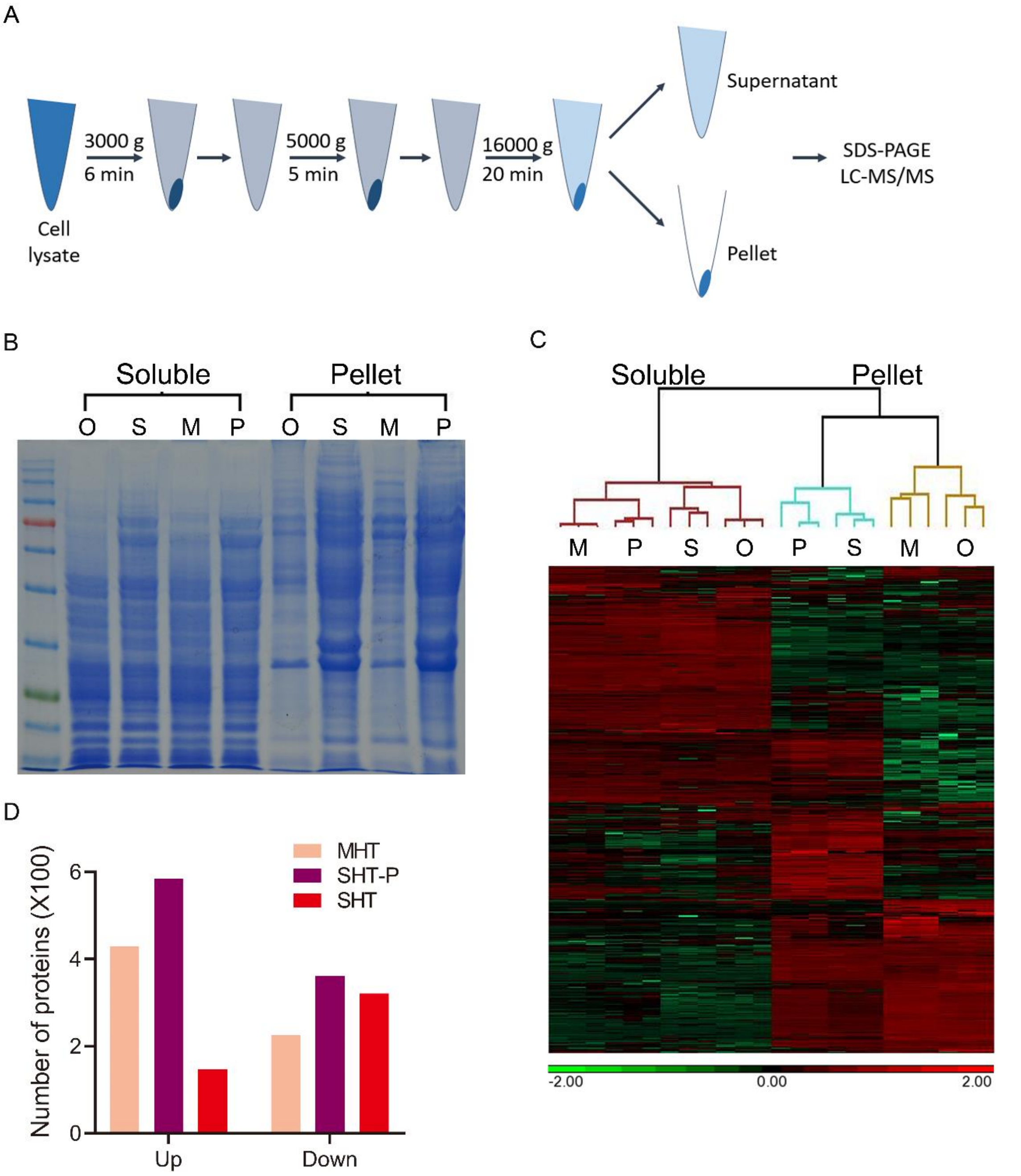

Fig S4. Proteomic data. (A) Workflow for the separation of soluble and pellet proteins. (B) SDS-PAGE analysis of Coomassie brilliant blue stained soluble (left four lanes) and pellet (right four lanes) proteins. O: OT; M: MHT; P: SHT-P; S: SHT. (C) Expression heatmap of all the proteins identified by proteomics analysis. Soluble and pellet proteins were analyzed by liquid chromatography with tandem mass spectrometry (LC-MS/MS). A total of 4,492 proteins were detected. O: OT; M: MHT; P: SHT-P; S: SHT. (D) Number of soluble proteins that were up- or downregulated (q-value < 0.05 and $|FC| \geq 2$) at MHT, SHT-P and SHT compared to OT.
