# Supplementary material for: Serine peptidases and increased amounts of soluble proteins contribute to heat priming of the plant pathogenic fungus Botrytis cinerea
Source: mBio. 2023 Jul 6;14(4):e01077-23. doi: 10.1128/mbio.01077-23 (PMC10470532; doi:10.1128/mbio.01077-23)
Supplement: Fig. S5 — Images showing the effects of STPs on priming. [file mbio.01077-23-s0005.pdf]

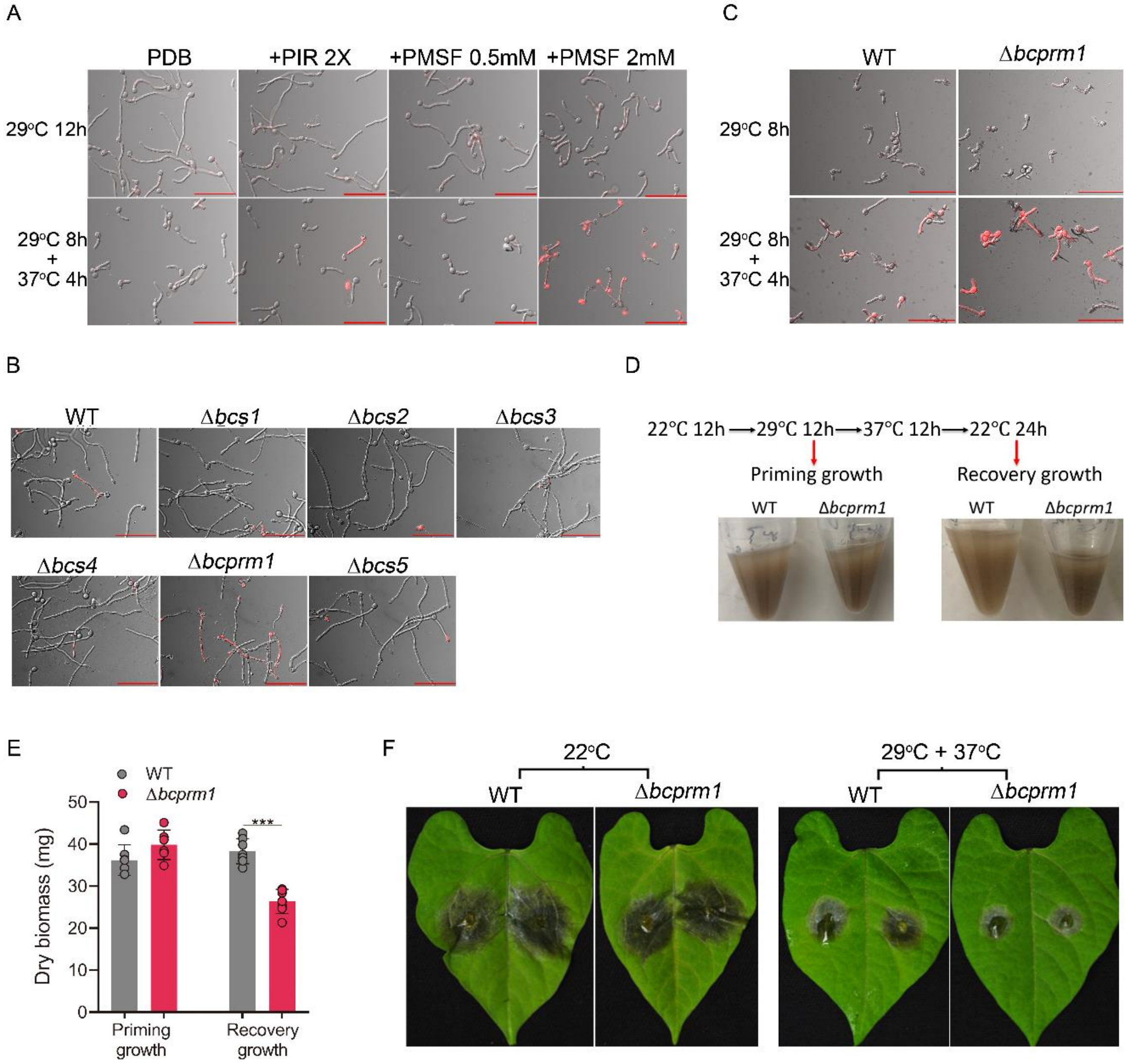

Fig S5. Images showing the effects of STPs on priming. (A-C) Combined DIC and fluorescence (rhodamine filter) microscopic images of GTs after staining with PI (A, B) or DiBAC4(5) (C). Δ*bcs1-5*: Δ*bcin_15g03150*, Δ*bcin_15g04670,* Δ*bcin_06g00330,* Δ*bcin_06g00620* and Δ*bcin_07g01720.* PIR: protease inhibitor. Scale bars, 100 µm. (D) Workflow of biomass measurement. (E) Measurement of dry biomass. Graph represents at least six biological replications with overlaid individual data points. Values are presented as the mean of replicates ± s.d. Statistical differences were determined according to unpaired two-tailed Student' t-test (****P* < 0.001). (F) Representative photographs of *P. vulgaris* leaves showing the effects of *bcprm1* deletion on priming pathogenicity under priming conditions.
