# Supplementary material for: Serine peptidases and increased amounts of soluble proteins contribute to heat priming of the plant pathogenic fungus Botrytis cinerea
Source: mBio. 2023 Jul 6;14(4):e01077-23. doi: 10.1128/mbio.01077-23 (PMC10470532; doi:10.1128/mbio.01077-23)
Supplement: Supplemental Tables — Tables S1, S2, S4 to S6, S10, and S11. [file mbio.01077-23-s0006.pdf]

Table S1. Summary of RNA-Seq experiment setup

Fungi materials were prepared from Spores under four treatment conditions. h: hour

Treatments	Temperatures			Biological replicates
	22°C	29°C	37°C	
Optimal Temperature (OT)	8h + 2h			4
Moderately High Temperature (MHT)		8h + 2h		4
Severely High Temperature-Priming (SHT-P)		8h	2h	4
Severely High Temperature (SHT)	8h		2h	4

Table S2. Summary of RNA-Seq data

Sample	Treatment	Total reads	Phred score	Mapped reads	Ratio of mapped reads
A1	22°C 10h	19903178	34.1946	19604233	0.9850
A2	22°C 10h	26015855	34.2152	25612874	0.9845
A3	22°C 10h	21766563	34.2198	21447687	0.9854
A4	22°C 10h	25840505	34.1587	25311628	0.9795
B1	22°C 8h+37°C 2h	22619396	34.1919	22256256	0.9839
B2	22°C 8h+37°C 2h	24221582	34.1558	23764020	0.9811
B3	22°C 8h+37°C 2h	24746542	34.1943	24312564	0.9825
B4	22°C 8h+37°C 2h	26729067	34.1522	26212289	0.9807
C1	29°C 10h	21875095	33.9702	21506157	0.9831
C2	29°C 10h	24627919	34.2077	24251656	0.9847
C3	29°C 10h	25793829	34.0608	25354266	0.9830
C4	29°C 10h	24028329	34.1311	23423548	0.9748
D1	29°C 8h+37°C 2h	29513826	34.2152	29043828	0.9841
D2	29°C 8h+37°C 2h	21712867	34.1088	21069460	0.9704
D3	29°C 8h+37°C 2h	26392593	34.2027	25869728	0.9802
D4	29°C 8h+37°C 2h	25655481	34.0693	25046932	0.9763

Table S4. GO enrichment results of 4814 DEGs (pFDR < 0.05, lFCl ≥2)

Upregulated DEGs

MHT vs OT

GO term	Description	P-value
GO:0008236	serine-type peptidase activity	1.99E-07
GO:0006508	proteolysis	1.33E-05
GO:0022857	transmembrane transporter activity	2.34E-05
GO:0000272	polysaccharide catabolic process	8.53E-05
GO:0055085	transmembrane transport	9.69E-05
GO:0004553	hydrolase activity, hydrolyzing O-glycosyl compounds	0.0007
GO:0005215	transporter activity	0.00122
GO:0004185	serine-type carboxypeptidase activity	0.00322
GO:0004252	serine-type endopeptidase activity	0.00421
GO:0004339	glucan 1,4-alpha-glucosidase activity	0.00485
GO:0016020	membrane	0.00585
GO:2001070	starch binding	0.00944
GO:0005975	carbohydrate metabolic process	0.01266
GO:0016021	integral component of membrane	0.01636
GO:0006865	amino acid transport	0.02235
GO:0008810	cellulase activity	0.02235
GO:0055114	oxidation-reduction process	0.03655
GO:0003777	microtubule motor activity	0.03954
GO:0007018	microtubule-based movement	0.03954
GO:0000774	adenyl-nucleotide exchange factor activity	0.04089
GO:0003997	acyl-CoA oxidase activity	0.04089
GO:0004342	glucosamine-6-phosphate deaminase activity	0.04089
GO:0004563	beta-N-acetylhexosaminidase activity	0.04089
GO:0005777	peroxisome	0.04089
GO:0006040	amino sugar metabolic process	0.04089
GO:0006635	fatty acid beta-oxidation	0.04089
GO:0009254	peptidoglycan turnover	0.04089
GO:0019310	inositol catabolic process	0.04089
GO:0050113	inositol oxygenase activity	0.04089
GO:0019441	tryptophan catabolic process to kynurenine	0.0495

SHT-P vs OT

GO term	Description	P-value
GO:0016788	hydrolase activity, acting on ester bonds	0.00182
GO:0008236	serine-type peptidase activity	0.0022
GO:0004523	RNA-DNA hybrid ribonuclease activity	0.00309
GO:0008131	primary amine oxidase activity	0.00309
GO:0009308	amine metabolic process	0.00309
GO:0008081	phosphoric diester hydrolase activity	0.00395
GO:0003824	catalytic activity	0.0087
GO:0003779	actin binding	0.01101
GO:0004813	alanine-tRNA ligase activity	0.02125

GO:0006044	N-acetylglucosamine metabolic process	0.02125
GO:0006419	alanyl-tRNA aminoacylation	0.02125
GO:0043039	tRNA aminoacylation	0.02125
GO:0048038	quinone binding	0.02455
GO:0016791	phosphatase activity	0.03037
GO:0006508	proteolysis	0.03083
GO:0071949	FAD binding	0.04819

SHT vs OT

**GO term**	**Description**	**P-value**
GO:0016491	oxidoreductase activity	6.41E-06
GO:0003824	catalytic activity	0.000137
GO:0004523	RNA-DNA hybrid ribonuclease activity	0.00177
GO:0006098	pentose-phosphate shunt	0.00177
GO:0008152	metabolic process	0.002298
GO:0010181	FMN binding	0.009299
GO:0016791	phosphatase activity	0.014345
GO:0004813	alanine-tRNA ligase activity	0.014666
GO:0006044	N-acetylglucosamine metabolic process	0.014666
GO:0006419	alanyl-tRNA aminoacylation	0.014666
GO:0009082	branched-chain amino acid biosynthetic process	0.014666
GO:0019904	protein domain specific binding	0.014666
GO:0019915	lipid storage	0.014666
GO:0043039	tRNA aminoacylation	0.014666
GO:0016788	hydrolase activity, acting on ester bonds	0.020141
GO:0006890	retrograde vesicle-mediated transport, Golgi to endopla	0.040456
GO:0016832	aldehyde-lyase activity	0.040456
GO:0008081	phosphoric diester hydrolase activity	0.047637

Downregulated DEGs

MHT vs OT

**GO term**	**Description**	**P-value**
GO:0008061	chitin binding	0.00019
GO:0016491	oxidoreductase activity	0.00074
GO:0005576	extracellular region	0.00788
GO:0071949	FAD binding	0.01165
GO:0004571	mannosyl-oligosaccharide 1,2-alpha-mannosidase activi	0.0144
GO:0016831	carboxy-lyase activity	0.01974
GO:0004553	hydrolase activity, hydrolyzing O-glycosyl compounds	0.02173
GO:0010181	FMN binding	0.02898
GO:0003906	DNA-(apurinic or apyrimidinic site) endonuclease activit	0.03248
GO:0004351	glutamate decarboxylase activity	0.03248
GO:0006536	glutamate metabolic process	0.03248
GO:0006825	copper ion transport	0.03248
GO:0006878	cellular copper ion homeostasis	0.03248

GO:0008535	respiratory chain complex IV assembly	0.03248
GO:0016799	hydrolase activity, hydrolyzing N-glycosyl compounds	0.03248
GO:0019629	propionate catabolic process, 2-methylcitrate cycle	0.03248
GO:0047547	2-methylcitrate dehydratase activity	0.03248
GO:0051726	regulation of cell cycle	0.03248
GO:0005975	carbohydrate metabolic process	0.03332
GO:0030248	cellulose binding	0.03682
GO:0016829	lyase activity	0.0475

SHT-P vs OT

**GO term**	**Description**	**P-value**
GO:0005840	ribosome	9.5E-30
GO:0003735	structural constituent of ribosome	1.7E-29
GO:0006412	translation	1.7E-29
GO:0005622	intracellular anatomical structure	2.9E-15
GO:0000786	nucleosome	2.4E-05
GO:0005852	eukaryotic translation initiation factor 3 complex	0.00182
GO:0015935	small ribosomal subunit	0.00182
GO:0003743	translation initiation factor activity	0.00362
GO:0005488	binding	0.00396
GO:0046982	protein heterodimerization activity	0.00836
GO:0006520	cellular amino acid metabolic process	0.00881
GO:0019843	rRNA binding	0.00881
GO:0005524	ATP binding	0.01069
GO:0006260	DNA replication	0.01196
GO:0006351	transcription, DNA-templated	0.01635
GO:0005815	microtubule organizing center	0.01904
GO:0006352	DNA-templated transcription, initiation	0.0298
GO:0016818	hydrolase activity, acting on acid anhydrides, in phosph[illegible]	0.0298
GO:0003723	RNA binding	0.0425
GO:0000030	mannosyltransferase activity	0.04271
GO:0005664	nuclear origin of replication recognition complex	0.04271
GO:0005681	spliceosomal complex	0.04271
GO:0006164	purine nucleotide biosynthetic process	0.04271
GO:0006400	tRNA modification	0.04271
GO:0008897	holo-[acyl-carrier-protein] synthase activity	0.04271
GO:0016763	transferase activity, transferring pentosyl groups	0.04271
GO:0017183	peptidyl-diphthamide biosynthetic process from peptid[illegible]	0.04271
GO:0031047	gene silencing by RNA	0.04271
GO:0031625	ubiquitin protein ligase binding	0.04271
GO:0051537	2 iron, 2 sulfur cluster binding	0.04271

SHT vs OT

**GO term**	**Description**	**P-value**
GO:0008270	zinc ion binding	0.008547
GO:0003777	microtubule motor activity	0.009203

GO:0007018	microtubule-based movement	0.009203
GO:0004386	helicase activity	0.015357
GO:0005815	microtubule organizing center	0.015357
GO:0016810	hydrolase activity, acting on carbon-nitrogen (but not p	0.015507
GO:0007165	signal transduction	0.016801
GO:0000723	telomere maintenance	0.025208
GO:0008234	cysteine-type peptidase activity	0.025208
GO:0015095	magnesium ion transmembrane transporter activity	0.025208
GO:0015693	magnesium ion transport	0.025208
GO:0030151	molybdenum ion binding	0.025208
GO:0000030	mannosyltransferase activity	0.037939
GO:0005938	cell cortex	0.037939
GO:0006974	cellular response to DNA damage stimulus	0.037939
GO:0008897	holo-[acyl-carrier-protein] synthase activity	0.037939
GO:0031047	gene silencing by RNA	0.037939
GO:0032065	maintenance of protein location in cell cortex	0.037939
GO:0046873	metal ion transmembrane transporter activity	0.038784
GO:0016887	ATPase activity	0.04259
GO:0046983	protein dimerization activity	0.045351

Table S5. Summary of cluster A and B genes (pFDR<0.05, FC ≥5)
GO:0008236, serine-type peptidase encoding genes

Cluster A: 39 genes

Gene ID	Gene name	Description	
BCIN_06g00620	Bctpp2	Peptidases_S53; Peptidase domain in the S53 family	GO:0008236
BCIN_09g02350		Peptidase_S10; Serine carboxypeptidase	
BCIN_06g00330		Peptidases_S53; Peptidase domain in the S53 family	GO:0008236
BCIN_15g04670	Bcser8	Peptidases_S53; Peptidase domain in the S53 family	GO:0008236
BCIN_07g01720		DAP2; Dipeptidyl aminopeptidase/acylaminoacyl peptidase	GO:0008236
BCIN_01g06210		Peptidase_S10; Serine carboxypeptidase	
BCIN_15g03150		Peptidases_S53; Peptidase domain in the S53 family	GO:0008236
BCIN_16g02770	Bcmp1	M35_deuterolysin_like; Peptidase M35 domain of deuterolysins and related	
BCIN_08g02390		Abhydrolase; alpha/beta hydrolases	GO:0008236
BCIN_14g00610	Bcpg2	Glyco_hydro_28; Glycosyl hydrolases family 28	
BCIN_02g08230		Abhydrolase; alpha/beta hydrolases	
BCIN_11g06440		GH31_N; N-terminal domain of glycosyl hydrolase family 31 (GH31)	
BCIN_08g00740		Sugar_tr; Sugar (and other) transporter	
BCIN_12g03830		Sugar_tr; Sugar (and other) transporter	
BCIN_15g04710		2A0304; amino acid permease (GABA permease)	
BCIN_11g00260		2A0304; amino acid permease (GABA permease)	
BCIN_06g03790		CESA_like; CESA_like is the cellulose synthase superfamily	
BCIN_04g04190		CBM20_glucoamylase; Glucoamylase	
BCIN_14g05500	Bcgod1	GMC_oxred_C; GMC oxidoreductase	
BCIN_14g00760		SurE; Survival protein SurE	
BCIN_12g01060		IDO; Indoleamine 2,3-dioxygenase	
BCIN_01g04310		OafA; Peptidoglycan/LPS O-acetylase OafA/YrhL	
BCIN_13g01270	Bcvvd1	PRK13558; bacterio-opsin activator	
BCIN_02g03050		MFS_1; Major Facilitator Superfamily	
BCIN_05g04840	Bcdur3	SLC5sbd_DUR3; Na(+)/urea-polyamine cotransporter DUR3	
BCIN_02g08920		p450; Cytochrome P450	
BCIN_07g01340		unknown	
BCIN_03g03200		unknown	
BCIN_15g04720		unknown	
BCIN_16g02480		unknown	
BCIN_02g07070		unknown	
BCIN_09g00460		unknown	
BCIN_01g06950		unknown	
BCIN_01g01570		unknown	
BCIN_06g01060		unknown	
BCIN_15g00630		unknown	
BCIN_06g01990		unknown	
BCIN_09g02150		unknown	
BCIN_15g03040		unknown	

Cluster B: 16 genes

BCIN_06g00620	Bctpp2	Peptidases_S53; Peptidase domain in the S53 family	GO:0008236

BCIN_09g02350		Peptidase_S10; Serine carboxypeptidase	
BCIN_06g00330		Peptidases_S53; Peptidase domain in the S53 family	GO:0008236
BCIN_15g04670	Bcser8	Peptidases_S53; Peptidase domain in the S53 family	GO:0008236
BCIN_07g01720		DAP2; Dipeptidyl aminopeptidase/acylaminoacyl peptidase	GO:0008236
BCIN_16g02770	Bcmp1	M35_deuterolysin_like; Peptidase M35 domain of deuterolysins and related [illegible]	
BCIN_11g06440		GH31_N; N-terminal domain of glycosyl hydrolase family 31 (GH31)	
BCIN_11g00260		2A0304; amino acid permease (GABA permease)	
BCIN_06g03790		CESA_like; CESA_like is the cellulose synthase superfamily	
BCIN_04g04190		CBM20_glucoamylase; Glucoamylase	
BCIN_14g05500	Bcgod1	GMC_oxred_C; GMC oxidoreductase	
BCIN_14g00760		SurE; Survival protein SurE	
BCIN_12g01060		IDO; Indoleamine 2,3-dioxygenase	
BCIN_02g07070		unknown	
BCIN_06g01990		unknown	
BCIN_09g02150		unknown	

Table S6. Summary of proteomic experiment setup

Treatments	Temperatures			Replicates	Sample
	22°C	29°C	37°C		
Optimal Temperature (OT)	14h + 2h			3	A (A1, A2, A3)
Moderately High Temperature (MHT)		14h + 2h		3	C (C1, C2, C3)
Severely High Temperature-Priming (SHT-P)		14h	2h	3	D (D1, D2, D3)
Severely High Temperature (SHT)	14h		2h	3	B (B1, B2, B3)

Table S10. GO enrichment of 355 soluble priming candidate proteins

GO term	Name	P-value	Benjamini
GO:0000030	mannosyltransferase activity	3.74E-10	1.23E-07
GO:0016758	transferase activity, transferring hexosyl groups	1.02E-07	1.69E-05
GO:0017171	serine hydrolase activity	6.39E-07	5.26E-05
GO:0008236	serine-type peptidase activity	6.394E-07	5.259E-05
GO:0016757	transferase activity, transferring glycosyl groups	1.34E-06	8.79E-05
GO:0015932	nucleobase-containing compound transmembrane transporter activi	4.08E-06	0.0001916
GO:1901505	carbohydrate derivative transmembrane transporter activity	4.08E-06	0.0001916
GO:0000295	adenine nucleotide transmembrane transporter activity	1.23E-05	0.000338
GO:0005346	purine ribonucleotide transmembrane transporter activity	1.23E-05	0.000338
GO:0015216	purine nucleotide transmembrane transporter activity	1.23E-05	0.000338
GO:0015215	nucleotide transmembrane transporter activity	1.23E-05	0.000338
GO:0015605	organophosphate ester transmembrane transporter activity	1.23E-05	0.000338
GO:0008514	organic anion transmembrane transporter activity	4.43E-05	0.0011205
GO:0004169	dolichyl-phosphate-mannose-protein mannosyltransferase activity	6.69E-05	0.0015726
GO:0004252	serine-type endopeptidase activity	9.81E-05	0.0021343
GO:0005347	ATP transmembrane transporter activity	0.0001038	0.0021343
GO:0015075	ion transmembrane transporter activity	0.0004316	0.0083526
GO:0008509	anion transmembrane transporter activity	0.0010155	0.0185602
GO:0008233	peptidase activity	0.0011542	0.0194533
GO:0016627	oxidoreductase activity, acting on the CH-CH group of donors	0.0011826	0.0194533
GO:0016780	phosphotransferase activity, for other substituted phosphate groups	0.0014405	0.0225677

Table S11. List of Oligonucleotides

Name	Sequence (5' - 3')	Purpose
2020-rt-for	GCTCGTGTTGACATGGCTGGTCGTGATTTG	Real time PCR primers
2020-rt-rev	CAGAGCTTCTCCTTGATATCACGGACG	Real time PCR primers
4650-rt-for	AAGCGAGGAGGGCTACTTCT	Real time PCR primers
4650-rt-rev	CGACTTGAGGAGTTCAGCGT	Real time PCR primers
0030-rt-for	GCGACGACATCAAAGAGTGC	Real time PCR primers
0030-rt-rev	CCTGGACCAAGTCTGGCAAT	Real time PCR primers
5710-rt-for	GTTACTCTTCTGGCCGCTGT	Real time PCR primers
5710-rt-rev	ATCGGTTGGAACGCTGCTAT	Real time PCR primers
2300-rt-for	GAAAGGTCGCGGAAGCAATC	Real time PCR primers
2300-rt-rev	TGCTTGCAACTCCACTGTCT	Real time PCR primers
5750-rt-for	CCGGGGTGATGAGTTTCGAT	Real time PCR primers
5750-rt-rev	TGTATGCTCTCTTCGACGGC	Real time PCR primers
1560-rt-for	ACCACCAACTCCCCTTCTCT	Real time PCR primers
1560-rt-rev	TTTGGAAGGACAAGGGAGGC	Real time PCR primers
3150-rt-for	CAGCACGAGGTGACTCTGTT	Real time PCR primers
3150-rt-rev	GAGTTCGGATAGCACCCTCG	Real time PCR primers
1720-rt-for	TCAAGCCACCACAACCAAGT	Real time PCR primers
1720-rt-rev	GTTGGGGGTGGAAATCACCT	Real time PCR primers
5' 3150 CRISPR	GAAGGAGAGCTTCCCCACTC	to knockout of BCIN_15g03150
3' 3150 CRISPR	ATACTGAGCCTCCTCAAATC	to knockout of BCIN_15g03150
5' 4670 CRISPR	GATTGATGATTGACTGATGG	to knockout of BCIN_15g04670
3' 4670 CRISPR	GCGGTTTAAGTGGTGTCACT	to knockout of BCIN_15g04670
5' 0330 CRISPR	GGTTTAACGTGCGGGGTTTG	to knockout of BCIN_06g00330
3' 0330 CRISPR	ACCGGACATGAGAGGTGTAG	to knockout of BCIN_06g00330
5' 0620 CRISPR	ATTCGACGTTGTGTGATCCG	to knockout of BCIN_06g00620
3' 0620 CRISPR	AGTATTGCGGCTTGGTGATA	to knockout of BCIN_06g00620
5' 2390 CRISPR	ACAGAGCTTCAGAGCCCTAG	to knockout of BCIN_08g02390
3' 2390 CRISPR	AGTTTACGAGCAGCTTGCTT	to knockout of BCIN_08g02390
5' 1720 CRISPR	AAAGGAAGGTGGGAGTGTCT	to knockout of BCIN_07g01720
3' 1720 CRISPR	AAACGAGTTCCTACTACTTG	to knockout of BCIN_07g01720
5' flank 3150 for	TCCATCTGGACTTTCCTTCAATGC	to confirm the deletion of BCIN_15g3150 by CRISPR
3' flank 3150 rev	GCTGTGTAAGGCAGAGAGGAG	to confirm the deletion of BCIN_15g3150 by CRISPR
5' flank 4670 for	AACATCGAGCCGTACTGTAGC	to confirm the deletion of BCIN_15g04670 by CRISPR
3' flank 4670 rev	TTACAGGTAGTCATGCGCGAG	to confirm the deletion of BCIN_15g04670 by CRISPR
5' flank 0330 for	TTCCCATCCCTTCACAACATTC	to confirm the deletion of BCIN_06g00330 by CRISPR
3' flank 0330 rev	TCCAGCAGTATCGCATCC	to confirm the deletion of BCIN_06g00330 by CRISPR
5' flank 0620 for	CAGTCCAGTCCCGATGATTAAG	to confirm the deletion of BCIN_06g00620 by CRISPR
3' flank 0620 rev	GTATTTCATTCTGCTCGTCTGG	to confirm the deletion of BCIN_06g00620 by CRISPR
5' flank 2390 for	CATCGTCCCAAGCTCTTTCC	to confirm the deletion of BCIN_08g02390 by CRISPR
3' flank 2390 rev	CCCAAGCTCTTCTCTGCATC	to confirm the deletion of BCIN_08g02390 by CRISPR
5' flank 1720 for	CATGTCGGAGTACGGATCAAG	to confirm the deletion of BCIN_07g01720 by CRISPR
3' flank 1720 rev	TATGCTGATGAAGGTGAAGGTG	to confirm the deletion of BCIN_07g01720 by CRISPR
